# A Novel Application of Ti-Substituted Polyoxometalates: Anti-Inflammatory Activity in OVA-Induced Asthma Murine Model

**DOI:** 10.1155/2016/3239494

**Published:** 2016-06-29

**Authors:** Dong Li, Xiuzhu Gao, Jingmin Gu, Yuan Tian, Yaqing Liu, Zheng Jin, Dongmei Yan, Ya-Guang Chen, Xun Zhu

**Affiliations:** ^1^Department of Immunology, College of Basic Medical Sciences, Jilin University, Changchun 130021, China; ^2^Department of Hepatology, First Hospital of Jilin University, Changchun 130021, China; ^3^College of Veterinary Medicine, Jilin University, Changchun 130062, China; ^4^Key Laboratory of Polyoxometalates Science of Ministry of Education, Faculty of Chemistry, Northeast Normal University, Changchun 130024, China

## Abstract

*Objective*. Asthma is a chronic inflammatory disorder. Despite extensive researches into the treatment and management of it, current treatments and management strategies are still limited. The search for a novel approach to its treatments is urgently needed. Researches on the potential medical use of polyoxometalates (POMs) have already shown it has antiviral and antitumor bioactivities. But the effects of POM in immune systems are still largely unknown.* Methods*. In order to investigate the role of POM in the asthmatic disease, we used OVA-induced asthma murine model and observed the pathological changes between mice that received three different Ti-substituted POMs (0.3 *μ*g per mouse per dose) when challenged with OVA. We also measured the type 2 cytokine expressions to reveal the potential mechanism.* Results and Conclusions*. Our results showed that two Ti-substituted POMs, K_5_H_2_[FeW_11_TiO_40_]·17H_2_O and K_5_H[H_2_ZnW_11_TiO_40_]·35H_2_O, could reduce OVA-induced lung inflammation, serum IgE level (around 2000 ng/mL to less than 1000 ng/mL), leukocytes infiltration in the lung, and cytokines levels (including IL-4, IL-5, IL-13, and TNF-*α*) but Ti-centered POM K_4_[TiW_12_O_40_]·10H_2_O did not. Thus, Ti-substituted POMs may have pharmaceutical values especially in treatments for asthmatic diseases.

## 1. Introduction

Polyoxometalates (POMs) are a kind of negatively charged inorganic substances constructed by early transitional metal ions such as vanadium (V), niobium (Nb), molybdenum (Mo), and tungsten (W). The most typical and fully studied POMs are those with Keggin anion structure, XM_12_O_40_^*n*−^, M = Mo, W; X = B, Al, Ga, Si, Ge, P, As, Ti, V, Cr, Fe, Co, Ni, Cu, Zn, and so forth; X is called as heteroatom or central atom, due to its relative stability and easy availability, as well as potential application in catalysis, medicine, and materials [1, 2]. One M atom in the anion may be replaced by other metal atoms, giving so-called monosubstituted POM. The metal substitution extends considerably the number of POMs and modified the properties of the anions. In this field, there are much reports about the substituted POM with main group elements such as Si and P as heteroatom, and those with transition elements as X were reported relatively less [3–5], although CoW_11_CoO_40_H_2_^8−^ was described in 1960s [6].

In the aspect of application in medicine of POMs, three types of POM bioactivities, antiviral, antitumor, and antibacterial, have been reported [7–11]. The third aspect developed later than the former two [12]. The POM with Keggin, lacunary Keggin, Wells-Dawson, double-Keggin, and Keggin-sandwich structures enhanced the antibacterial activity of *β*-lactam antibiotics on methicillin-resistant* Staphylococcus aureus* [12, 13]. The mechanism of the synergistic effect by the POM is discussed in terms of the depression of penicillin-binding protein 20 (PBP20) coded by mecA gene [14]. On the other hand, in bioactivity study of substituted POMs, only several Ti-substituted POMs with Keggin (or Keggin-sandwich) structure exhibit obvious antiviral and antitumor activity [15–18]. Titanium seems to be an important metal ion to afford antiviral action of POM in mice [17] and titanium-containing POMs were less toxic for actively growing cells than their vanadium containing counterparts [15]. Thus, titanium-containing POM may be safe and potent for therapeutic use in* in vivo* virus infections. Up to date, there are few reports about the study on antibacterial or anti-inflammation activity of Ti-substituted POM.

Asthma is major thread in public health as it affects more than 300 million people worldwide [19, 20]. Asthma, especially allergic allergen induced asthma, is associated with airway eosinophilic inflammation and increased serum IgE level [21, 22]. Although there were extensive experimental and human studies about the mechanisms of asthmatic airway inflammation and remodeling, more work is still required to fully understand the mechanisms of asthmatic diseases [23]. At present, it is generally believed that the type 2 helper T cell (Th2) cytokines (including interleukin- (IL-) 4, IL-5, and IL13), also known as type 2 cytokines since the major source of them are Th2 cells, and type 2 innate lymphoid cells [24] play an essential role in the pathogenesis of asthma [25, 26].

Although asthma symptoms can be controlled in most of the patients by current standard therapies, for example, inhaled corticosteroids like *β*2-adrenergic receptor agonists and oral leukotriene inhibitors [27], there are still around 10% of patients that are not responsive to these treatments, as they still suffer from bronchial hyperresponsiveness and limitations in airflow. Furthermore, those patients with uncontrolled asthma have higher risk of morbidity and mortality rate [28], and they require the largest share of economic resources and healthcare systems. Therefore, the search for additional therapeutic approaches is urgently needed. In this work we used the OVA-induced asthma murine model to investigate the effect and mechanisms of Ti-substituted POM in asthmatic diseases.

## 2. Materials and Methods

### 2.1. Chemicals and Instruments

All reagents were commercially available and used without any previous purification. Elemental analyses of W, Zn, Fe, and Ti were carried out with a Leeman ICP spectrometer. Thermogravimetric analyses were carried out by using a NETZSCH STA 449 F3 instrument, with a heating rate of 10°C·min^−1^ under a nitrogen atmosphere. The IR spectra in KBr pellets were recorded in the range of 400–4000 cm^−1^ with a Magna-560 FT/IR spectrophotometer. The UV spectra were recorded on a TU1810 ultraviolet-visible spectrophotometer in aqua solution.

### 2.2. Synthesis

K_4_[TiW_12_O_40_]·10H_2_O (TiW): Na_2_WO_4_·2H_2_O(36.3 g, 0.11 mol) was dissolved into 100 mL of distilled water; then glacial acetic acid was added until the pH of the solution reached 6.3. The solution was incubated at 60°C for 0.5 h. Afterwards, 20 mL solution containing 4.8 g Ti(SO_4_)_2_ (pH = 1.0, 0.02 mol) was added under stirring. The resulting solution was then heated at 80–90°C for 4h. After the reaction solution was cooled to room temperature, 7.5 g solid KCl (0.1 mol) was added to the solution. The oil phase was formed while the solution was cooled to 4°C, and the solution was kept at 4°C; four days later the oil solidified and the separated solid was treated with warm water. The TiW yield was 20%. Element analysis calculation values for H_20_K_4_O_50_TiW_12_ (Mw = 3230.6): K 4.84%, Ti 1.48%, W 68.29% and H_2_O 5.57%; found values K 4.62%, Ti 1.37%, W 69.1% and H_2_O 6.5%. IR (in KBr pellet, cm^−1^): 3433 (s), 1629 (m), 964 (s), 882 (m), 796 (m), 741 (m), 425 (m).

K_5_H_2_[FeW_11_TiO_40_]·17H_2_O (FeWTi) was prepared as previously described [3] and checked by IR spectroscopy and thermogravimetric analysis. Element analysis calculation values for FeH_36_K_5_O_57_TiW_11_ (Mw = 4512.59): K 6.0, Fe 1.7%, Ti 1.5%, W 61.9% and H_2_O 9.9%; found: K 6.2%, Fe 1.8%, Ti 1.4%, W 62.1% and H_2_O 10.2%. IR (KBr pellet, cm^−1^): 3433 (s), 1629 (m), 933 (s), 872 (m), 7769 (m), 717 (m), 425 (m).

K_5_H[H_2_ZnW_11_TiO_40_]·35H_2_O (ZnWTi) was prepared as previously described [4] and checked by IR spectroscopy and thermogravimetric analysis. Element analysis calculation values for H_73_K_5_O_75_TiW_11_Zn (Mw = 3604.64): K 5.42%, Ti 1.33%, Zn 1.81%, W 56.1% and H_2_O 18.0%; found: K 5.32%, Ti 1.37%, Zn 1.91%, W 56.81% and H_2_O 17.9%. IR (KBr, pellet, cm^−1^): 3445 (s), 1631 (m), 933 (s), 867 (m), 757 (m), 690 (m).

### 2.3. Stability of the Compounds in Aqua Solution

Keggin structure anions of polyoxotungstates have a characteristic absorption band at about 260 nm in their UV spectra [4]. The intensity of this band of monovacant or monosubstituted Keggin structure anions becomes lower but still can be used for identification of the anions. The absorption curves of three compounds in fresh and stock (0.01 mol·L^−1^) aqua solutions of pH = 4–7 were recorded in 200–400 nm. The maximum wavelengths for absorbance are TiW 259 nm, ZnWTi 256 nm, and FeWTi 257 nm. The absorption bands of the anions are unchanged in the range of pH = 5–7, under which the bioactivity experiments are carried out. The solutions of three compounds with pH = 6 gave the same absorption curves after 7 days.

### 2.4. Mice

Specific-pathogen-free male C57BL/6 mice weighing 18 to 22 g (purchased from Beijing Weitonglihua Laboratory Animal Co., Ltd., Beijing, China) were housed in specific-pathogen-free conditions at Jilin University, China. All experiments were performed in accordance with the National Guidelines for Experimental Animal Welfare and with approval of the Animal Welfare and Research Ethics Committee at Jilin University.

### 2.5. Enzyme-Linked Immunosorbent Assay

The concentrations of cytokines in BAL fluid and IgE in serum were determined by enzyme-linked immunosorbent assay (ELISA, eBioscience, Inc., CA, US) according to manufacturers' instructions as described previously [29, 30].

### 2.6. OVA-Induced Mice Asthma Model

Mice were given i.p. injections with 100 mg of OVA (ovalbumin, Sigma-Aldrich, MO, US) in 2% alum (Aluminium Hydroxide Gel Adjuvant, Brenntag, Denmark) and then challenged i.n. on days 8, 9, and 10 with 10 mg of OVA or PBS as described previously [31, 32]. POMs (0.3 *μ*g per mouse per dose) were injected to mice on days 8, 9, and 10 as the same time with OVA or PBS together. Mice were sacrificed 48 h after the last challenge by the i.p. injection of 500 *μ*L of Avertin (20 mg/mL 2,2,2-tribromoethanol, Sigma-Aldrich, MO, US). Bronchoalveolar lavage fluid (BALF) was collected and analyzed as previously described [29].

### 2.7. Quantitative PCR

RNA was purified from tissue samples using the RNeasy Mini Kit following the manufacturer's instructions as described previously (Qiagen, UK) [29, 30]. Reverse Transcription (RT) of RNA into cDNA was carried out using High-Capacity cDNA Reverse Transcription Kits (Applied Biosystems, CA, US). Real-time polymerase chain reaction (RT-PCR) was performed using Fast SYBR Green Master Mix on a Prism 7900HT (Applied Biosystems, CA, US). The primers used were as follows:* Il4*, forward 5′-CAT GGC TTG GGT ACA GGT CT-3′, reverse 5′-TTT GTA GTG GGA GGG GAC AG-3′;* Il5*, forward 5′-GAA GTG TGG CGA GGA GAG AC-3′, reverse 5′-GCA CAG TTT TGT GGG GTT TT-3′;* Il13*, forward 5′-GAA TCC AGG GCT ACA CAG AAC-3′, reverse 5′-AAC ATC ACA CAA GAC CAG ACT C-3′;* Ifng*, forward 5′-ACT TTG CTT CTG CCT TTC CA-3′, reverse 5′-ACA AGG TCA CCC ACA GGA-3′;* Tnf*, forward 5′-TCC CCT TCA TCT TCC TCC TT-3′, reverse 5′-CAT GCG TCC AGC TGA CTA AA-3′.

### 2.8. Histologic Analysis

The larger left lung lobe was excised, fixed in 4% buffered formalin, and embedded in paraffin. Sections (4 mm) were stained with hematoxylin and eosin (CellPath, Newtown, UK) [29]. The pathology score (from 1–4) was determined by using the method modified from a previous report [33], briefly, a numerical value was assigned based on the number of inflammatory cell infiltrate layers around the blood vessels and airways (1, no cells; 2, <3 cell layers; 3, 4–9 cell layers; 4, >10 cell layers).

### 2.9. Bone Marrow-Derived Mast Cells

Bone marrow-derived MC (BMMC) were generated from 7–9-week-old mice by isolating bone marrows from femurs. Cells were then cultured in IMDM (Invitrogen, MA, US) supplemented with 10% FBS, nonessential amino acids, 50 mM 2-ME (Sigma-Aldrich, MO, US), and 3 ng/mL IL-3 (PeproTech, NJ, US) for 4 weeks.

### 2.10. Degranulation Assays

For assessment of MC degranulation, degranulation assays were performed. Briefly, 1 × 10^6^/mL BMMC were loaded with 1 *μ*g/mL anti-DNP IgE (Sigma-Aldrich, MO, US) in culture medium without IL-3 overnight. Then, cells were washed three times in Modified Tyrode's Buffer (135 mM NaCl, 5 mM KCl, 1 mM MgCl_2_, 10 mM HEPES, 5.6 mM dextrose, and 0.1% (w/v) BSA) and then triggered for 60 min at 37°C with stimulation buffer (5 nM PMA (Sigma-Aldrich, MO, US) with 2 *μ*M ionomycin (Sigma-Aldrich, MO, US)) at 4 × 10^6^ cells/mL. To evaluate the inhibition effects of polyoxometalate, 1–100 *μ*g/mL WTi, FeWTi, or ZnWTi was also added in the stimulation buffer, respectively. After stimulation, the concentration of histamine in supernatants was measured by ELISA (Immundiagnostik AG, Bensheim, Germany). And the inhibition rate was calculated by dividing the concentration of histamine in supernatants from mast cells that received POMs by the concentration of histamine in supernatants from mast cells that did not receive POMs.

### 2.11. Statistical Analysis

Analysis between* in vivo* groups was examined by Mann-Whitney *U* test or ANOVA followed by Student's *t*-test. All data are expressed as means ± SEM. Values of *p* < 0.05 were considered significant.

## 3. Results

### 3.1. Polyoxometalates FeWTi and ZnWTi Attenuate OVA-Induced Lung Inflammation

**Figure 1:**
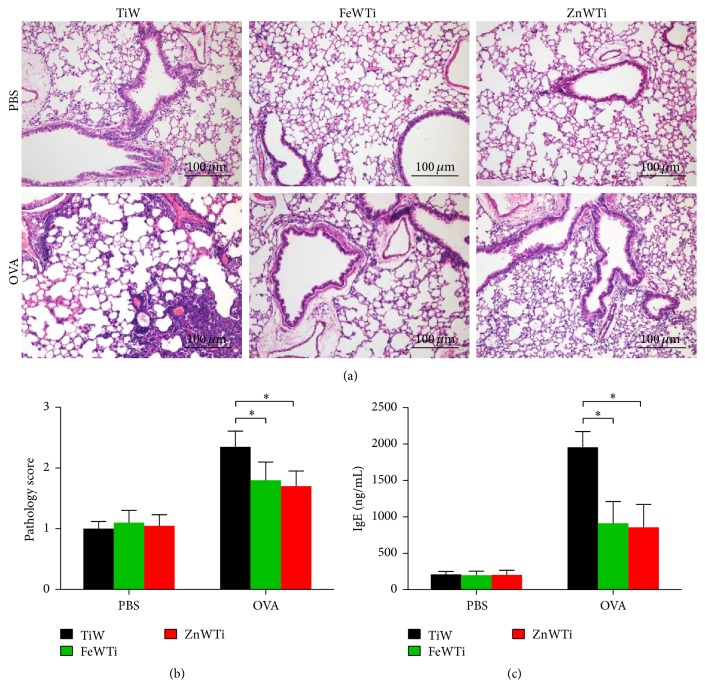
Polyoxometalates FeWTi and ZnWTi reduce OVA-induced lung inflammation. (a) Lung H&E staining; (b) lung pathology score; (c) serum IgE level measured by ELISA; bars = SEM; *n* = 7 mice/group/experiment; scale bar = 100 *μ*m; ^*∗*^ *p* < 0.05 compared to mouse treated with TiW. Data are representative of 3 experiments.

Given the well-established contribution of POM in the activation, proliferation, or suppression of immune cells, we reasoned that certain POMs like K_4_H[H_2_FeW_11_TiO_40_]·17H_2_O (FeWTi) or K_5_H[H_2_ZnW_11_TiO_40_]·35H_2_O (ZnWTi) but not TiW may reduce OVA-induced asthma in mice. To evaluate the effect of these chemicals in asthma, mice were sensitized with OVA plus adjuvant or PBS on day 1; then the mice were challenged with OVA or PBS for three times on days 8, 9, and 10; finally all the mice were sacrificed 48 hours after the last challenge, and FeWTi, ZnWTi, or TiW (0.3 *μ*g per mouse) were injected intranasally at the same time with challenges. Mice that received POM alone could not develop lung inflammation, but in OVA sensitized and challenged groups, mice that received FeWTi or ZnWTi had significantly less severe lung inflammation compared to mice that received TiW (Figures 1(a) and 1(b)). FeWTi or ZnWTi treatment was also with lower IgE serum levels compared with TiW groups (Figure 1(c)).

### 3.2. Polyoxometalates FeWTi and ZnWTi Reduce Inflammatory Cells Infiltrations

**Figure 2:**
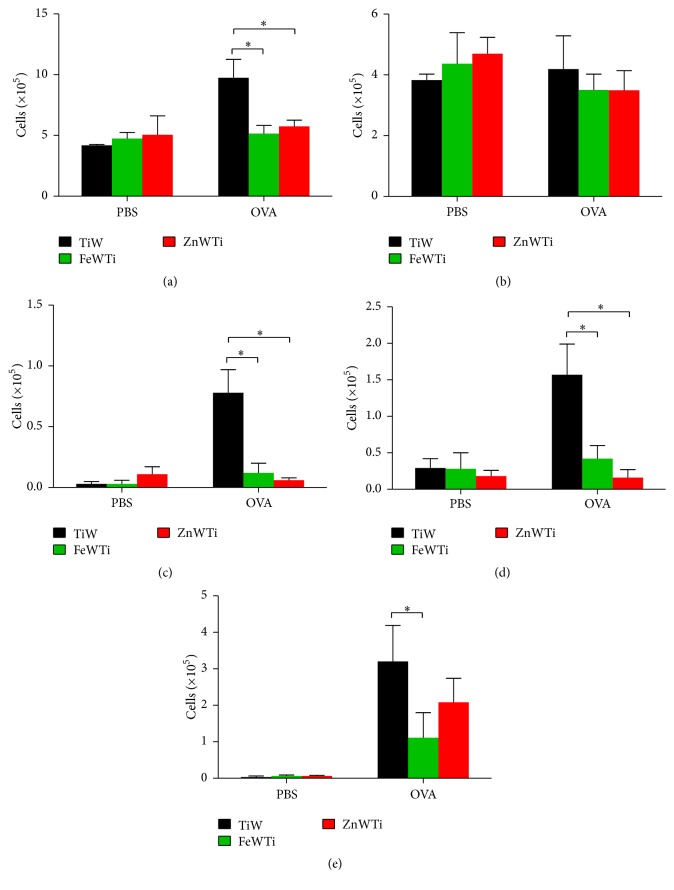
Polyoxometalates FeWTi and ZnWTi reduce inflammatory cells infiltration in the airway. Cells in the BALF were stained and counted; numbers of (a) total cells, (b) macrophages, (c) neutrophils, (d) lymphocytes, and (e) eosinophils; bars = SEM; *n* = 7 mice/group/experiment; ^*∗*^ *p* < 0.05 compared to mouse treated with TiW. Data are representative of 3 experiments.

After we established that FeWTi or ZnWTi treatment contributed to less severe lung inflammation in OVA-induced asthma mouse model, we next investigated the inflammatory cell infiltration in the airway by performing cellular analysis on the bronchoalveolar lavage fluid (BALF). Compared with TiW treated mice, FeWTi or ZnWTi treated mice showed decreased inflammatory cell infiltration in the BALF (Figure 2(a)), especially neutrophils. Lymphocyte and eosinophil numbers are significantly reduced in FeWTi or ZnWTi treated mice than in TiW treated mice (Figures 2(c) and 2(e)), but the number of macrophages is not significantly different among groups (Figures 2(b) and 2(d)).

### 3.3. Polyoxometalates FeWTi and ZnWTi Reduce Proinflammatory Cytokines in the BALF

**Figure 3:**
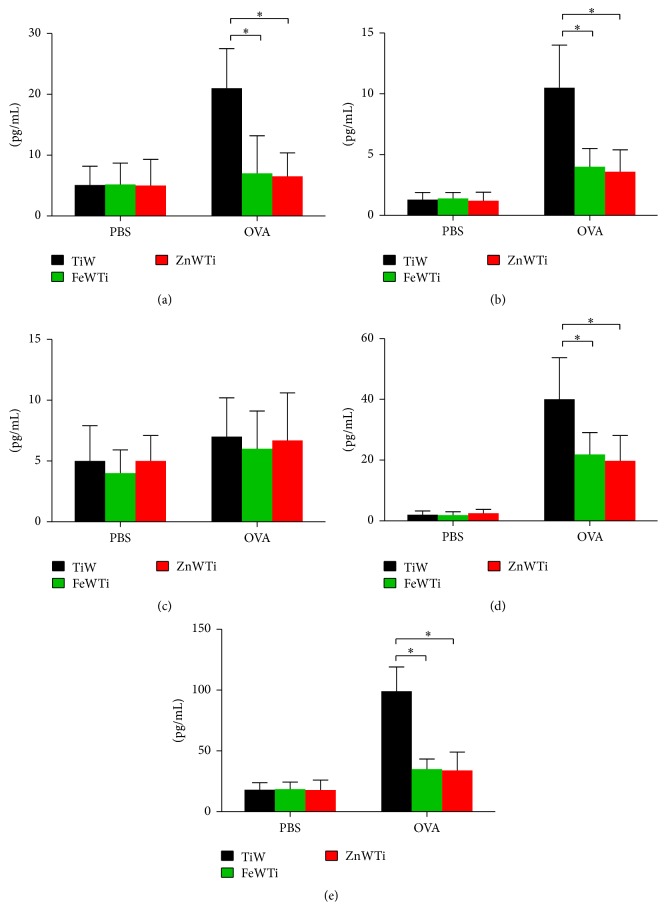
Polyoxometalates FeWTi and ZnWTi reduce inflammatory cytokines profile in OVA-induced asthma. Cytokines in the BALF were measured by ELISA; the concentration of (a) IL-4, (b) IL-5, (c) IFN-*γ*, (d) IL-13, and (e) TNF-*α*; bars = SEM; *n* = 7 mice/group/experiment; ^*∗*^ *p* < 0.05 compared to mouse treated with TiW. Data are representative of 3 experiments.

After we established that FeWTi or ZnWTi treatment contributed to less severe lung inflammation and cell infiltration in OVA-induced asthma mouse model, we next investigate the mechanisms of this phenomenon by analyzing the cytokine profiles of BAL fluid. The levels of type 2 cytokines (IL-4, IL-5, and IL-13) and TNF-*α* are significantly lower in FeWTi or ZnWTi treated mice compared to those in TiW treated mice, but the level of type 1 cytokine IFN-*γ* is not significantly different (Figure 3).

### 3.4. Polyoxometalates FeWTi and ZnWTi Reduce mRNA Gene Expression of Proinflammatory Cytokines

**Figure 4:**
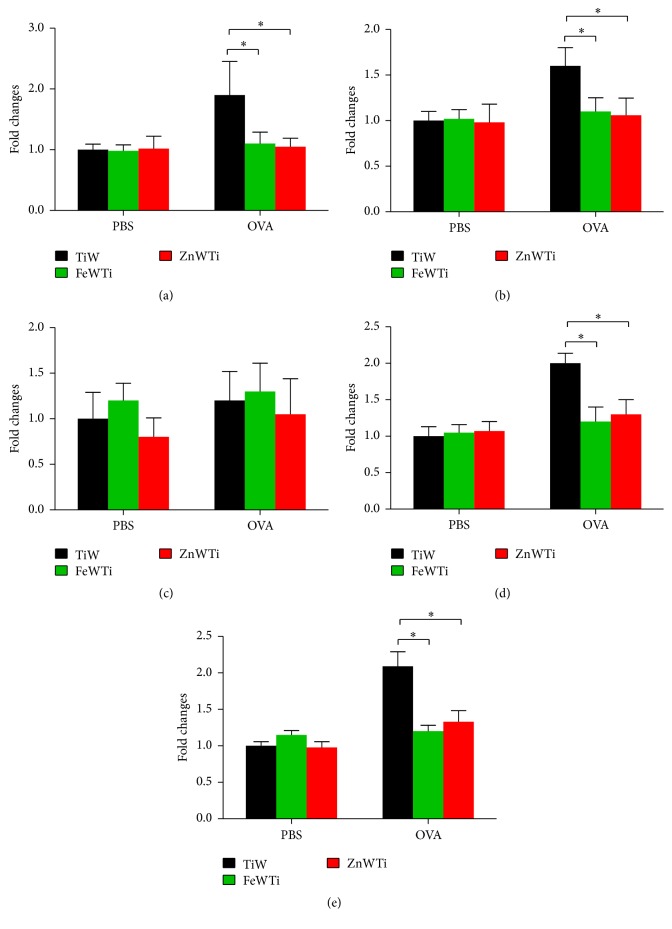
Polyoxometalates FeWTi and ZnWTi reduce mRNA expression of inflammatory cytokines in OVA-induced asthma. The mRNA expressions of cytokines of lung tissues were measured by q-PCR; the expression of (a)* Il4*, (b)* Il5*, (c)* Ifng*, (d)* Il13*, and (e)* Tnf*; bars = SEM; *n* = 7 mice/group/experiment; ^*∗*^ *p* < 0.05 compared to mouse treated with TiW. Data are representative of 3 experiments.

Next, in order to identify the sources of cytokines which were reduced by POM treatments, we measured the mRNA expression profile of lung tissue. The results had similar trend with cytokine profile; type 2 cytokines like* Il4*,* Il5*, and* Il13* and* Tnf* but not type 1 cytokine like* Ifng* are significantly lower in FeWTi or ZnWTi treated mice. These results indicate that inflammatory cells that infiltrated in the lung might cause the higher concentration of type 2 cytokines which was observed in the BAL fluid (Figure 4).

## 4. Discussion

**Figure 5:**
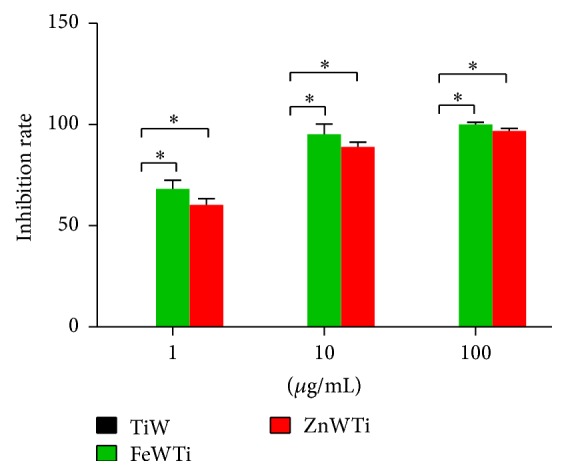
Polyoxometalates FeWTi and ZnWTi suppress the degranulation of mast cells. Mast cells were stimulated by ionomycin and PMA with or without different doses of TiW, FeWTi, or ZnWTi for 1 hour; the concentrations of histamine in supernatant were measured by ELISA. The inhibition rate was calculated by comparing the histamine expression of mast cells treated with different chemicals to mast cells treated with completed media. ^*∗*^ *p* < 0.05 compared to mouse treated with TiW. Data are representative of 3 experiments.

In short, out work showed that using Ti-substituting POMs like FeWTi and ZnWTi could reduce inflammation and inflammatory cell infiltration in OVA-induced asthma murine model. This was also companied by the reduced proinflammatory cytokine production. To investigate the antiasthma potential of these two chemicals, we also tested their effect on mast cells, which played essential role in the asthmatic reactions. Both FeWTi and ZnWTi could significantly reduce the degranulation of mast cells but not TiW (Figure 5).

Asthma is still a major health threat despite decades of researches into it. And asthma exacerbation related hospitalization and readmission remain an unsolved issue. One of the reasons for this is *β*-adrenoceptor tolerance induced by repeated high dose usage [34]. The use of anti-inflammatory drugs is considered to be very effective initially, but their endocrine effects were proven to be controversial later [35, 36]. Recent approaches in the treatment of asthma were focused on the secondary consequences of asthma instead of the underlying mechanisms and aetiological causes of asthma, such as targeting mast cell, suppressing its activation directly or indirectly via neutralizing IgE, or using histamine agonist. Target T helper cells, cytokines, or cytokine receptors are also considered, although their involvement in asthma is well established, but all these efforts have achieved limited success [37]. This may be due to the fact that asthma cannot be considered as a homogeneous disorder; different subtypes of asthma have already been identified. Therefore, more researches into the mechanisms of asthma and reagents with therapeutic value are required.

POMs have already been shown to have great potential of pharmaceutical value, as they have already been proven to have anticancer [12, 38] and antivirus [7, 39, 40] activities. In the reported results [10–14], titanium seems to be an important metal ion to afford antiviral action of polyoxotungstate in mice [17] and titanium-containing POMs are less toxic for actively growing cells than their vanadium containing counterparts [15]. Thus, titanium-containing POM may be safe and potent for therapeutic use in * in vivo* virus infections. In this work we used three titanium-containing POMs to investigate their antiasthma effects. The results showed that both titanium-substituted FeWTi and ZnWTi could reduce inflammation and inflammatory cell infiltrations in OVA-induced asthma murine model but TiW did not.

**Figure 6:**
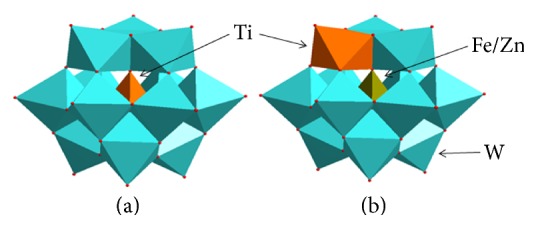
Keggin anion structure. (a) TiW_12_O_40_^4−^ and (b) Fe(Zn)W_11_TiO_40_^5−/6−^.

Why does not TiW have the exact antiasthma effects as FeWTi and ZnWTi do? The main reason is the site of the Ti atom in the anion. Three anions all have the Keggin anion structure (Figure 6). In TiW anion Ti atom as a heteroatom locates in the centre of anion and is surrounded by four triads of tungsten-oxo octahedra (Figure 6(a)). So this Ti atom does not contact with other materials in the environment. In FeWTi and ZnWTi anions the heteroatoms are Fe atom and Zn atom; Ti atom substitutes one of twelve W atoms (Figure 6(b)). Ti atom shares oxygen atoms with four W atoms and Fe or Zn atoms; its sixth coordination site may be occupied by a substitutable water molecule or other groups. The surrounding of the substituted atom (transition metal, here is Ti atom) is so similar to those of transition metal atom in porphyrin that the monosubstituted Keggin anion is called inorganic porphyrin analogue [41, 42]. That is, Ti atom in the periphery of the anion has high reactivity.

To sum up, this work provides further evidences in the pharmaceutical potentials of Ti-substituted polyoxometalates. Particularly for asthmatic diseases, further researches into the mechanisms and potent side effects of this type of chemicals may provide novel therapeutic strategy.

## Additional Points

(1) Ti-substituted polyoxometalates attenuate inflammations in an asthmatic setting. (2) Ti-substituted polyoxometalates reduce the production of type 2 cytokines like IL-4, IL-5, and IL-13.
